# ﻿*Iugisporipsathyrareticulopilea* gen. et sp. nov. (Agaricales, Psathyrellaceae) from tropical China produces unique ridge-ornamented spores with an obvious suprahilar plage

**DOI:** 10.3897/mycokeys.90.85690

**Published:** 2022-06-22

**Authors:** Sheng-Nan Wang, Yu-Guang Fan, Jun-Qing Yan

**Affiliations:** 1 Jiangxi Key Laboratory for Conservation and Utilization of Fungal Resources, Jiangxi Agricultural University, Nanchang, Jiangxi 330045, China; 2 Key Laboratory of State Forestry Administration on Forest Ecosystem Protection and Restoration of Poyang Lake Watershed, Jiangxi Agricultural University, Nanchang, Jiangxi 330045, China; 3 Institute of Edible Mushrooms, Fujian Academy of Agricultural Sciences; National and Local Joint Engineering Research Center for Breeding & Cultivation of Features Edible Mushrooms, Fuzhou 350011, China; 4 Key Laboratory of Tropical Translational Medicine of Ministry of Education, Hainan Medical University, Haikou 571199, China; 5 Hainan Provincial Key Laboratory for Research and Development of Tropical Herbs, School of Pharmacy, Hainan Medical University, Haikou 571199, China

**Keywords:** Basidiomycete, fungal phylogeny, taxonomy

## Abstract

*Iugisporipsathyra*, a new psathyrelloid genus from tropical red soil of China, is established with *I.reticulopilea* as the type species. The new genus is characterised by basidiomata psathyrelloid, pileus rugose to appearing reticulate ridged, covered by persistent, but inconspicuous villus, pleurocystidia absent and ridge-ornamented spores with an obvious suprahilar plage. The genus is unique amongst Psathyrellaceae in producing ridge-ornamented spores with an obvious suprahilar plage and forms a distinct lineage within Psathyrellaceae, based on the Maximum Likelihood and Bayesian Inference analyses of a combined three-gene sequence dataset (ITS, LSU and β-*tub*). Full descriptions and photographs of the new genus and species are presented.

## ﻿Introduction

The Psathyrellaceae Vilgalys, Moncalvo & Redhead was established in 2001, based on the type genus *Psathyrella* (Fr.) Quél. by Vilgalys and Redhead (Redhead et al. 2001). More than 1300 names within the family, including synonyms and subspecies, are listed in Index Fungorum (http://www.indexfungorum.org). Species of Psathyrellaceae are cosmopolitan and often grow on decaying logs, woody debris, humus or soil, in woodlands, lawns or bogs and can have either broad or specific substrate relationships (Kirk et al. 2008).

Traditionally, the family included two types of species: psathyrelloid species and coprinoid species. During the classic period of morphological research, Fries (1838) classified the psathyrelloid species to AgaricusL.trib.Psathyrella Fr. Quélet (1872) promoted this group to the rank of genus. *Psathyrella* was finally accepted after the transfer of *Drosophila* Quél. species and emendations by Singer (1951,^1975^). Subsequently, Kits van Waveren (1985) removed the species with warty spores from *Psathyrella* and treated these as the genus *Lacrymaria* Pat. Although the boundaries of the genus were disputed, most researchers agreed that the psathyrelloid species should be classified in Coprinaceae R.Heim ex Pouzar subfamily Psathyrelloideae (Kuhner) Singer (Hawksworth et al. 1983; Kirk et al. 2001). During this same period, the coprinoid species were classified in *Coprinus* Pers. (Coprinaceae subfamily Coprinoideae Henn.) (Hawksworth et al. 1995; Kirk et al. 2001). *Coprinus* was circumscribed by Persoon (1797). However, Fries (1821) did not recognise the genus in his monograph *Systema Mycologicum* and classified the species in *Agaricus*. However, in his subsequent monograph *Epierisis systematis Mycologici*, Fries discarded his previous classification and again placed the coprinoid species in the independent genus *Coprinus* (Fries, 1838).

Although morphological studies provide abundant support for recognition of Psathyrellaceae, morphological data are inadequate to conclusively resolve the systematic relationships amongst the constituent genera and species. When the works of Hopple and Vilgalys (1999) and Redhead et al. (2001) were published, it became apparent that molecular biology techniques would profoundly alter the classical systematics of psathyrelloid species and coprinoid species. Based on these studies, *Coprinus* was split into four genera (*Coprinellus* P.Karst., *Coprinopsis* P.Karst., *Coprinus* and *Parasola* Redhead, Vilgalys & Hopple) (Redhead et al. 2001), restraining the generic name *Coprinus* to a small group centred on the type species *Coprinucomatus* (O.F.Müll.) Pers., which is now classified in the Agaricaceae Chevall. The other three genera, together with *Psathyrella* and *Lacrymaria*, were incorporated into the newly-established Psathyrellaceae. In 2015, *Psathyrella*, as a paraphyletic group, was also split, with the establishment of the segregate genera *Cystoagaricus* Singer emend. Örstadius & E.Larss., *Homophron* (Britzelm.) Örstadius & E.Larss., *Kauffmania* Örstadius & E.Larss. and *Typhrasa* Örstadius & E.Larss. (Örstadius et al. 2015). In 2020, *Candolleomyces* D.Wächt. & A.Melzer, *Britzelmayria* D.Wächt. & A.Melzer and *Olotia* D.Wächt. & A.Melzer were separated from *Psathyrella*, *Punjabia* D.Wächt. & A.Melzer and *Tulosesus* D.Wächt. & A.Melzer were separated from *Coprinellus*, *Narcissea* D.Wächt. & A.Melzer was segregated from *Coprinopsis* and *Hausknechtia* D.Wächt. & A.Melzer was erected for *Galerellafloriformis* Hauskn. (Wächter and Melzer 2020). *Heteropsathyrella* T.Bau & J.Q.Yan was established in 2021, based on the new species *He.macrocystidia* T.Bau & J.Q.Yan (Bau and Yan 2021a). Thus, the main systematic framework of Psathyrellaceae has been confirmed. In addition, *Ozonium* Link and *Hormographiella* Guarro & Gené, formerly members of the Psathyrellaceae, were established to accommodate the conidial anamorphs of certain species, now classified in *Coprinellus* (Nagy et al. 2013). *Gasteroagaricoides* D.A.Reid and *Macrometrula* Donk & Singer, two genera that, to date, have not been included in phylogenetic analyses, are retained in the Psathyrellaceae. There were 19 genera, in total, in the Psathyrellaceae before the new taxon we discovered was added.

From 2015, we initiated a study of Chinese psathyrelloid species and described 15 new taxa (Yan and Bau 2017, 2018a,b; Yan et al. 2019; Bau and Yan 2021a,b; Wang et al. 2021). By chance, we collected a psathyrelloid species with a reticulate-ridged pileus, that was reminiscent of *Pluteusthomsonii* (Berk. & Broome) Dennis, on the roadside in tropical China. After examining the micromorphology of the specimens, we observed that it produced ridge-ornamented spores with an obvious suprahilar plage. Surprisingly, phylogenetic analysis of molecular data revealed that it belonged to the Psathyrellaceae. Although abundant genera and species are recognised in the Psathyrellaceae, the majority of species have smooth spores. Verrucous spores have been observed only in *Lacrymaria*. Rough spores have been observed in *Coprinopsis*, *Coprinellus* and *Psathyrella*, but are extremely rare. Thus, the specimens are unique amongst Psathyrellaceae in producing ridge-ornamented spores with an obvious suprahilar plage. On the basis of our morphological and phylogenetic analyses, the specimens are described herein as a new species and a new genus is erected to accommodate the new species.

## ﻿Materials and methods

### ﻿Morphological studies

**Figure 1.**
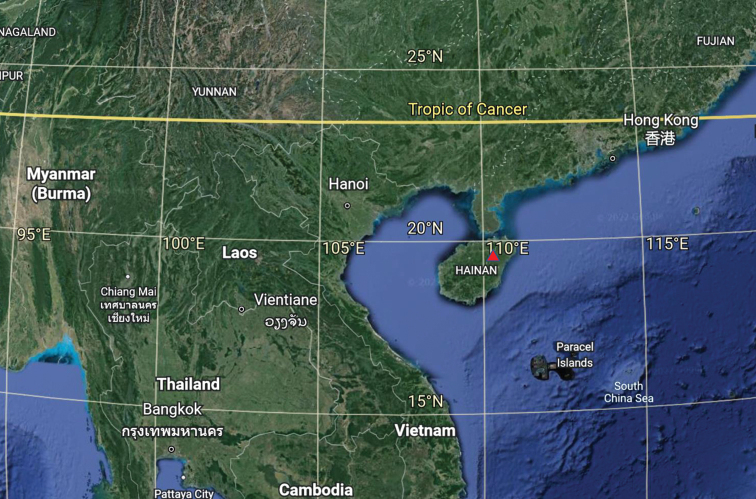
Map showing the location of the collection site of the specimens (red triangle).

Macroscopic descriptions and habitat details were based on detailed field notes of fresh basidiomata and photos. The location of the collection point is marked on the map (Fig. 1). Colour codes follow the Methuen Handbook of Colour (Kornerup and Wanscher 1978). Microscopic structures were observed and measured from dried specimens mounted in water, 5% potassium hydroxide (KOH), 10% ammonium hydroxide (NH4OH) or Melzer’s Reagent. Congo red was used as a stain when necessary (Horak 2005). A minimum of 100 basidiospores, basidia and cystidia from seven basidiomata (three collections) were randomly measured using an Olympus BX53 microscope. Detailed observations of spores were made by SEM. The measurements and Q values are recorded as (a)b–c(d), in which “a” is the lowest value, “b–c” covers a minimum of 90% of the values and “d” is the highest value. “Q” stands for the ratio of length and width of a spore (Bas 1969; Yu et al. 2020). Specimens were deposited in the Herbarium of Fungi, Jiangxi Agricultural University (HFJAU).

### ﻿DNA extraction and sequencing

DNA was extracted from dried specimens with the NuClean Plant Genomic DNA kit (CWBIO, China) (Ge et al. 2021; Na et al. 2022). Three regions (ITS, LSU and β-*tub*) were selected for the study and were amplified using the primer pairs by ITS1/ITS4 (White et al. 1990), LR0R/LR7 (Hopple and Vilgalys 1999) and B36f/B12r (Nagy et al. 2011), respectively. PCR was performed using a touchdown programme for all regions: 5 min at 95 °C; 1 min at 95 °C; 30 s at 65 °C (add -1 °C per cycle); 1 min at 72 °C; cycle 15 times; 1 min at 95 °C; 30 s at 50 °C; 1 min at 72 °C; cycle 20 times; 10 min at 72 °C (Bau and Yan 2021a). The sequencing was performed by Qing Ke Biotechnology Co. Ltd. (Wuhan City, China).

### ﻿Data analyses

A total of 221 nucleotide DNA (ITS, LSU and β-*tub*) sequences representing 93 taxa were used in subsequent analyses. Details are presented in Table 1. Some species of Agaricaceae, Mythicomycetaceae Vizzini, Consiglio & M. Marchetti and Nidulariaceae Dumort. were chosen as outgroup taxa according to the results of Zhao et al. (2017) and Vizzini et al. (2019). ITS, LSU and β-*tub* sequence datasets were separately aligned on the MAFFT online server (Katoh et al. 2019). Bayesian Inference (BI) and Maximum Likelihood (ML) phylogenetic analyses of the aligned concatenated dataset were respectively carried out in MrBayes v.3.2.7a and IQTREE v.2.1.2 (Nguyen et al. 2014) via the CIPRES web portal. For the BI analyses, optimal evolutionary models were selected using PartitionFinder2 (Lanfear et al. 2017) with the greedy algorithm and the AICc criterion. Four Monte Carlo Markov chains were run for 2 million generations, sampling every 100^th^ generation, with the first 25% of trees discarded as burn-in (Ronquist et al. 2012). For the ML analysis, models of sequence evolution were assessed in IQ-Tree prior to the analysis. The ML analysis was conducted using the ultrafast bootstrap option with 1,000 replicates and allowing partitions to have different seeds (--p). A nexus file contains alignment sequence and original tree of ML and Bayes is deposited in Suppl. material 1.

**Table 1.** Sequences used in this study.

Taxon	Voucher	ITS	LSU	β-*tub*
*Britzelmayriamultipedata*	LÖ237-04	KC992888	KC992888	KJ664867
*B.supernula*	LÖ250-04	KC992867	KC992867	KJ664849
*Candolleomyceseurysporus*	GLM-F126263 Type	MT651560	MT651560	MW369460
*C.subcacao*	HMJAU37807 Type	MW301064	MW301092	MW314063
*C.subminutisporus*	HMJAU37801 Type	MW301066	MW301094	MW314065
*C.subsingeri*	HMJAU37913 Type	MG734725	MW301098	MW314068
*Coprinellusandreorum*	CS1247 Type	MW621497	MW621007	–
*C.aureogranulatus*	CBS973.95	GQ249274	GQ249283	GQ249258
*C.aureogranulatus*	CBS753.96 Isotype	MH862611	–	–
*C.curtus*	NL-2339	FM878016	FM876273	FN396281
*C.deminutus*	NL-0761	JN159572	JN159592	JN159636
*C.disseminatus*	NL-2337	FM878017	FM876274	FN396282
*C.domesticus*	NL-1292	FN396102	HQ847132	FN396330
*C.silvaticus*	LÖ172-08	KC992943	KC992943	KJ664911
*Coprinopsisbabosiae*	NL-4139 Type	FN396128	FN396177	FN396352
*C.calospora*	CBS612.91 Type	GQ249275	GQ249284	GQ249259
*C.cortinatus*	NL-1621	FN396121	FN396171	FN396346
*C.musae*	JV06-179 Type	KC992965	KC992965	KJ664920
*C.musae*	JV06-180	KC992966	KC992966	KJ664921
*C.semitalis*	CBS291.77 Type	GQ249278	GQ249287	GQ249262
*C.udicola*	AM1240 Type	KC992967	KC992967	KJ664922
*C.villosa*	NL-1758 Type	JN943128	JQ045877	HQ847173
*Cystoagaricushirtosquamulosa*	Ramsholm800927	KC992945	KC992945	–
*C.olivaceogrisea*	WK8/15/63-5 Type	KC992948	KC992948	–
*C.silvestris*	LÖ191-92	KC992949	KC992949	–
*C.squarrosiceps*	Laessoe44835	KC992950	–	–
*C.strobilomyces*	E.Nagasawa9740	AY176347	AY176348	–
*Hausknechtiafloriformis*	WU22833 Type	JX968254	JX968371	–
*Heteropsathyrellamacrocystidia*	HMJAU37803	MW405101	MW413358	–
*H.macrocystidia*	HMJAU37802 Type	MW405102	MW413359	MW410997
*Homophroncamptopodum*	1997/956	KC992956	KC992956	–
*H.cernuum*	LÖ134-98	DQ389726	DQ389726	KJ664915
*H.crenulata*	W-K8/10/64-5 Type	KC992957	–	–
*H.spadiceum*	Enderle Epitype	DQ389729	DQ389729	–
*Iugisporipsathyrareticulopilea*	HFJAU1352 Type	ON207138	ON207137	ON210974
*I.reticulopilea*	HFJAU3181	ON207139	–	ON210975
*I.reticulopilea*	HFJAU3182	ON207140	–	ON210976
*Kauffmanialarga*	LÖ223-90	DQ389694	DQ389694	KJ664912
*K.larga*	LAS97-054	DQ389695	DQ389695	–
*Lacrymariaglareosa*	LAS06-019	KC992954	KC992954	KJ664914
*L.hypertropicalis*	Guzman29585 Type	KC992958	KC992958	KJ664916
*L.lacrymabunda*	EL70-03	DQ389724	DQ389724	–
*L.pyrotricha*	CBS573	GQ249280	GQ249289	GQ249264
*L.rigidipes*	LAS00-081	KC992953	KC992953	KJ664913
*L.subcinnamomea*	Smith16957 Type	KC992951	KC992951	–
*Narcisseacordispora*	SFSUDEH2073	AY461827	–	–
*N.cordispora*	LÖ41-01	DQ389723	–	KJ664910
*N.patouillardi*	NL-1687	FM878009	FM876265	FN396257
*Olotiacodinae*	GLM-F112430 Type	MG696611	MG674714	–
*Parasolaauricoma*	NL-0087	JN943107	JQ045871	FN396252
*P.conopilea*	LÖ186-02 Neotype	DQ389725	DQ389725	–
*P.kuehneri*	Ulje31-V-1987 Type	KY928608	KY928633	–
*P.lactea*	NL-0466	FM163192	FM160717	FN396254
*P.misera*	NL-0280 Neotype	FM163210	FM160699	–
*P.ochracea*	NL-3621 Type	JN943134	JQ045875	–
*P.parvula*	CAL1667 Type	NR_160509	NG064556	–
*P.plicatilis*	NL-0295	FM163216	FM160693	FN396253
*P.plicatilis*	NL-0075a Epitype	NR_171786	NG075167	–
*P.psathyrelloides*	CAL1753 Type	MK682756	MK682754	–
*Psathyrellaamygdalinospora*	HMJAU37952 Type	MW405104	MW413361	MW410991
*P.amygdalinospora*	HMJAU57044	MW405105	–	–
*P.fagetophila*	LÖ210-85 (M) Type	KC992902	KC992902	KJ664879
*P.fennoscandica*	HMJAU37918	MG734723	MW413365	MW410993
*P.fennoscandica*	LÖ484-05 Type	KC992903	KC992903	KJ664881
*P.noli-tangere*	LÖ83-03 Neotype	DQ389713	DQ389713	KJ664890
*P.seminuda*	Smith34091 (MICH) Type	KC992907	KC992907	–
*P.warrenensis*	Smith70162 (MICH) Type	KC992906	KC992906	–
*Punjabiapakistanica*	MEL2382843	KP012718	KP012718	–
*P.pakistanica*	LAH35323 Type	MH366736	–	–
*Tulosesuscanistri*	Walleyn877 Isotype	HQ846985	–	HQ847142
*T.cinereopallidus*	NL-0177 Type	HQ847001	HQ847090	HQ847149
*T.fuscocystidiatus*	NL-2720 Type	HQ846977	HQ847064	HQ847152
*T.hiascens*	NL-2536	FM878018	FM876275	FN396284
*T.pseudoamphithallus*	Ulje1288 Type	HQ846973	HQ847059	–
*T.radicellus*	NL-3168 Type	GU227719	HQ847077	GU227737
*T.sassii*	NL-1495	FN396101	FN396155	FN396329
*Typhrasagossypina*	Schumacher024	KC992946	KC992946	–
*T.nanispora*	Barta980706 Type	KC992947	KC992947	–
*T.polycystis*	HFJAU1454 Type	MW466538	MW466544	–
*T.rugocephala*	HFJAU1467 Type	MW466541	MW466546	–
**Outgroup**				
*Coprinuscomatus*	AFTOL_ID_626	AY854066	AY635772	–
*Crucibulumlaeve*	REGCrul1/DSH96-02	DQ486696	AF336246	–
*Cyathusstriatus*	DSH96-028/Cyst1/DSH96-001	DQ486697	AF336247	–
*Lepiotacristata*	ZRL20151133	LT716026	KY418841	–
*Leucocoprinusfragilissimus*	ZRL20151466	LT716029	KY418844	–
*Lycoperdonericaeum*	ZRL20151498	LT716030	KY418845	–
*Macrolepiotadolichaula*	xml2013058	LT716021	KY418836	–
*Mycocaliadenudata*	AFTOL2018/CBS494.85	DQ911596	DQ911597	–
*Mythicomycescorneipes*	AFTOL-ID972	DQ404393	AY745707	–
*M.corneipes*	KB51	KY648897	–	–
*Nidulaniveotomentosa*	AFTOL1945/CBS250.84	DQ917654	DQ986295	–
*Stagnicolaperplexa*	AH25260 Holotype	MK351609	MK353793	–
*S.perplexa*	AH25282 Paratype	MK351610	MK353794	–

## ﻿Results

### ﻿Phylogenetic analysis

Based on the BLAST results, the new species were found sharing less than 90.82% (ITS), 97.66% (LSU) and 87.03% (β-*tub*) similarity with the known species. The aligned concatenated dataset comprised 2,591 characters (ITS 835 bp, LSU 1338 bp and β-*tub* 418 bp), of which 983 sites were variable and 757 were parsimony informative. The best-fit evolutionary models used for the phylogenetic analyses were as follows: for the BI analysis, GTR + I + G for ITS and LSU and TIM + I + G for β-*tub*; and for the ML analysis, TIM2 + F + I + G4 for ITS, GTR + F + R4 for LSU and HKY + F + I + G4 for β-*tub.* The log-likelihood of the ML consensus tree was –27426.323 and the average standard deviation of split frequencies was less than 0.01 after 1,115,000 generations in the BI analysis. In the resulting trees, clades with a Bayesian posterior probability (BI-PP) ≥ 0.95 and ML bootstrap support (ML-BP) ≥ 75% were considered to be well supported.

**Figure 2.**
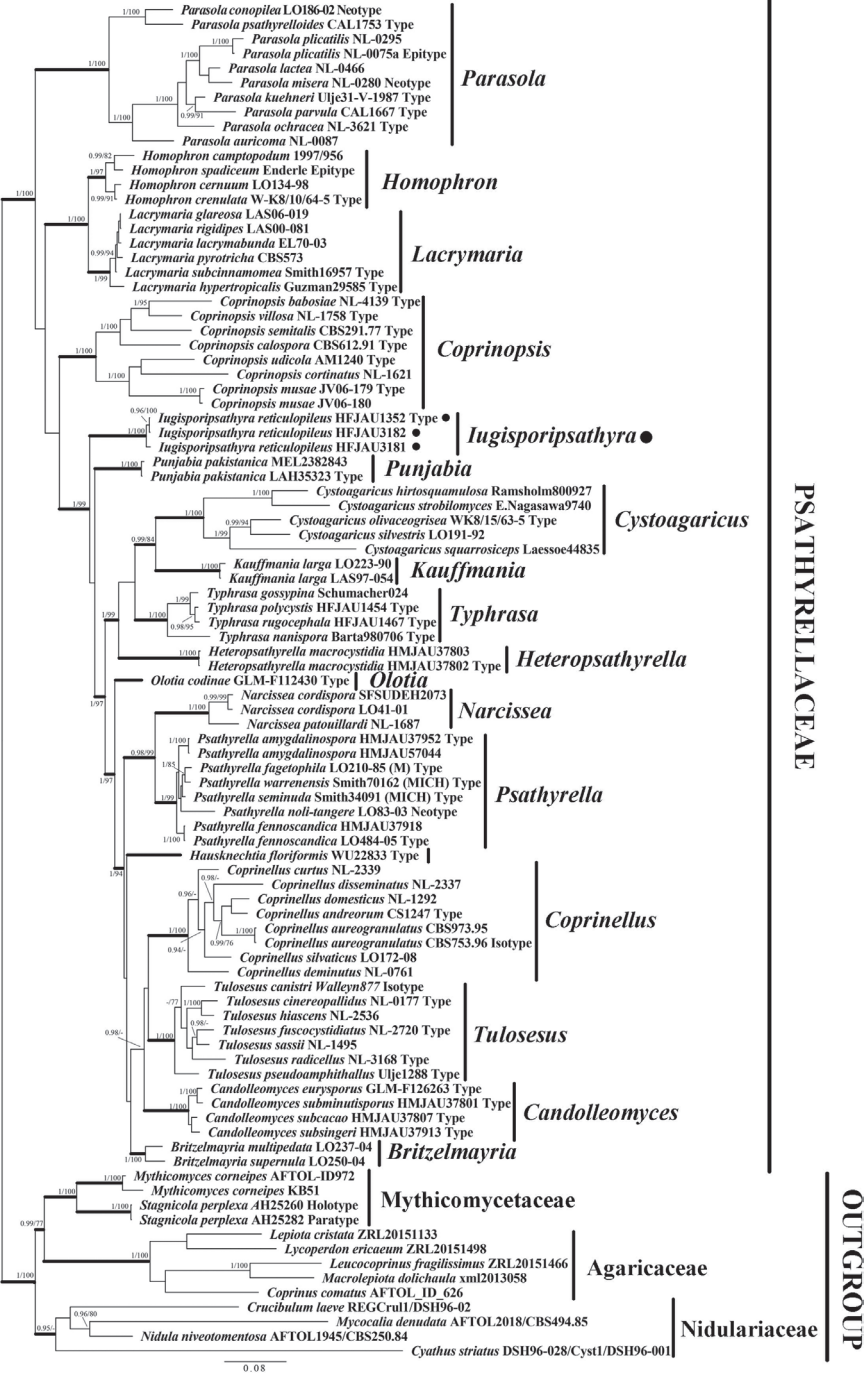
Phylogeny generated by Bayesian Inference, based on a concatenated sequence dataset for three nuclear DNA regions (ITS, LSU and β-*tub*). The tree was rooted with Agaricaceae spp., Mythicomycetaceae spp. and Nidulariaceae spp. Bayesian Inference posterior probabilities (BI-PP) ≥ 0.95 and Maximum Likelihood bootstrap percentages (ML-BP) ≥ 75% are shown as PP/BP at relevant nodes. (black circle) indicates newly-described taxa.

As shown in the BI tree in Fig. 2, all taxa of Psathyrellaceae formed a well-supported monophyletic lineage (BI-PP = 1; ML-BP = 100%). Within Psathyrellaceae, 18 major supported clades with a high statistical support value (BI-PP ≥ 0.95, ML-BP ≥ 75%) represented a total of 17 (out of 19) known genera and a new genus. *Iugisporipsathyra* formed a distinct lineage (BI-PP = 1; ML-BP = 100%) clearly separated from currently recognised genera.

### ﻿Taxonomy

#### ﻿ Iugisporipsathyra

Taxon classificationFungiAgaricalesPsathyrellaceae

J.Q. Yan, Y.G. Fan & S.N. Wang gen. nov.

3F06F9DF-4D54-56D4-94DA-8EB9ED2BF448

843734

##### Etymology.

Iugi-, iugis (Latin), ridge; -spori-, sporis (Latin), spores; Iugispori-, refers to its spore ornamentation; -psathyra, one of the synonyms of *Psathyrella*, refers to its similarity to *Psathyrella*.

##### Description.

Basidiomata psathyrelloid, fragile, non-deliquescent. Pileus hygrophanous, rugose to appearing reticulate ridged, covered by persistent and inconspicuous villus. Lamellae adnexed, brown. Stipe white, central, hollow. Spores amygdaliform in profile view, ovoid to elongate in face view, inamyloid, brown, fades in concentrated sulphuric acid, ridged and rarely verrucose ornamentation, suprahilar plage obvious. Basidia monomorphic. Pseudoparaphyses abundant. Pleurocystidia absent. Cheilocystidia present. Pileipellis hymeniderm, pyriform cell mixed with simple hairs.

##### Type species.

*Iugisporipsathyrareticulopilea* J.Q. Yan, Y.G. Fan & S.N. Wang

##### Notes.

The combination of veil absent, pleurocystidia absent and spores ornamented with ridges or rarely verrucose, with an obvious suprahilar plage is unique in Psathyrellaceae.

#### ﻿ Iugisporipsathyra reticulopilea

Taxon classificationFungiAgaricalesPsathyrellaceae

J.Q. Yan, Y.G. Fan & S.N. Wang sp. nov.

0E3EF4D4-73C8-54DA-99EA-4CAA3C9FDC86

843801

Fig. 3

##### Etymology.

reticulo-, reticular; reticulopilea, referring to the surface characteristic of the pileus.

##### Description.

Pileus 30–90 mm broad, oblate when young, expanding to plane, surface dry, rugose to appearing reticulate ridged, hygrophanous, pale yellow to greyish-yellow (4A3–4B2), becoming yellowish-white (4A2) as pileus dries, centre and ridged area darker, brown to dark brown (7D6–7F6), becoming greyish-yellow (4B2) as pileus dries. Pileus surface covered by inconspicuous villus. Villus very short, white (4A2), persistent. Veil absent. Context 3.0–4.0 mm broad, fragile, dirty white (7A1–7B2). Lamellae 3.5–10 mm broad, crowded, adnexed, 2–3 tiers of lamellulae, dirty white (7A1–7B2), becoming brown (7E6–7E8) as spores mature, edge white (7A1–7B1) and saw-toothed under 20× magnification. Stipe 50–80 mm long, 3.0–10 mm thick, fragile to fibrous, white to dirty white (7A1–7B1), cylindrical, hollow, gradually thickening towards base, 8.0–17 mm thick at base. Stipe surface covered with small, white, evanescent fibrils.

**Figure 3.**
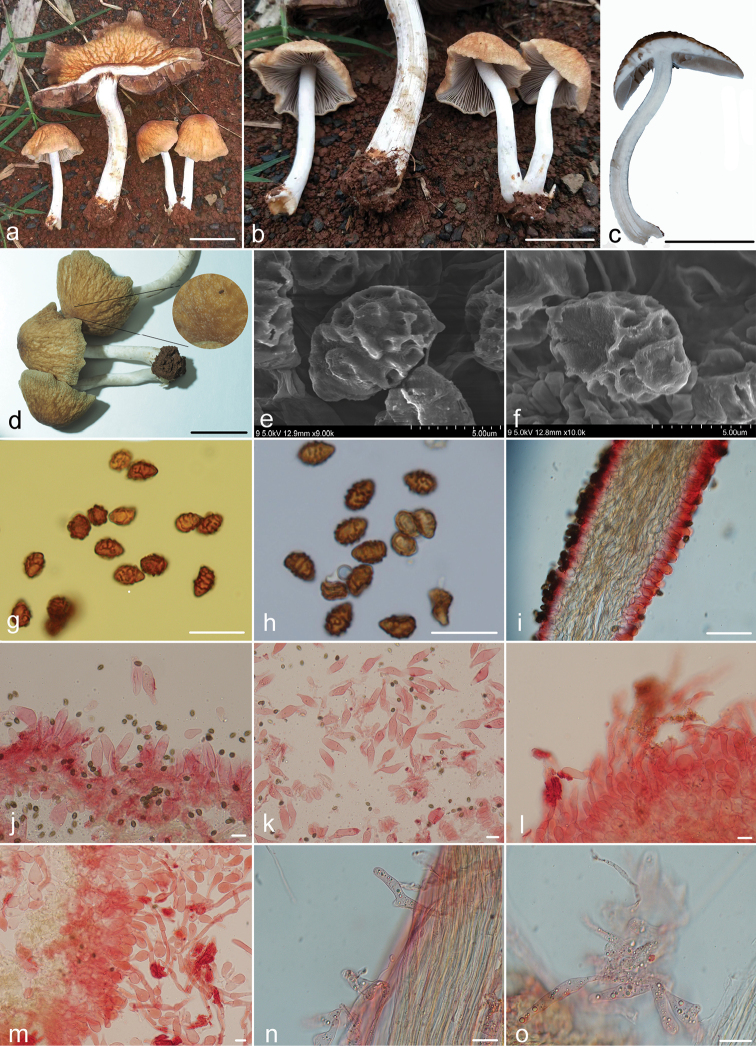
Macroscopic and microscopic structures of *Iugisporipsathyrareticulopilea***a–d** Basidiomata **e, f** spores viewed by scanning electron microscopy **g** spores in Melzer’s Reagent **h** spores in water **i** hymenophore **j, k** cheilocystidia **l, m** pileipellis and hairs hyphae **n, o** caulocystidia. Scale bars: 20 mm (**a–d**); 20 μm (**g–o**). Structures of **i–o** were observed in 5% KOH solution and Congo red was used as the stain.

Spores (7.5–)8.0–9.7(–10.5) × (4.0–)4.5–6.0 μm, Q = 1.5–2.0, amygdaliform in profile view, (4.5–)4.8–6.0(–6.3) μm broad, ovoid to elongate in face view, inamyloid, red-brown in water, brown in alkaline solution, fades in concentrated sulphuric acid, ornamentation up to 1.0 μm high, composed of irregular ridges and rarely verrucose, variable in length, partly connected, sometimes forming a zebroid pattern or closed meshes, suprahilar plage obvious, germ pore absent. Basidia (19–)22–29 × 9.5–12.0 μm, clavate, hyaline, 4- or 2-spored. Pseudoparaphyses abundant. Pleurocystidia absent. Cheilocystidia (37–)40–61(–68) × (9.5–)12–18(–22) μm, hyaline, utriform with obtuse to broadly obtuse apex, base tapering to a short or long stipe. Caulocystidia 50–90 × 6.0–14 μm, scattered or caespitose, various, mostly narrow clavate, hyaline. Trama of gills subparallel. Pileipellis hymeniderm, composed of a 1-cell-deep layer of pyriform cells, mixed with sparsely simple hairs, pyriform cells (35–)38–60 (–62) × (12–)14–23 μm, hairs hyphae, separate, 7.0–10 μm broad. Clamps present.

##### Known distribution.

Tropical China (Hainan Province).

##### Habit and habitat.

Scattered or 2–3 caespitose on red soil of roadside under broadleaf tree.

##### Specimens examined.

China. Hainan Province, Ding’an County, Longhu Town, 2 Jan 2019, Yu-Guang Fan, Jun-Qing Yan HFJAU 1352 (holotype); 4 Jan 2019, Jun-Qing Yan, Sheng-Nan Wang, HFJAU 3181, HFJAU 3182.

##### DNA sequence of type.

ON207138 (ITS), ON207137 (LSU), ON210974 (β-*tub*).

##### Notes.

Differs from other species in Psathyrellaceae by having ridge-ornamented spores with an obvious suprahilar plage.

## ﻿Discussion

The discovery of *I.reticulopilea* has transformed our traditional understanding of Psathyrellaceae. The species is unique amongst Psathyrellaceae in producing ridge-ornamented spores with an obvious suprahilar plage. This feature is so unusual that it seems difficult to associate it with Psathyrellaceae. However, the characteristic of the spores of fading in concentrated sulphuric acid is in common with other species in this family (Singer 1986; Kirk et al. 2008; Padamsee et al. 2008; Nagy et al. 2013; Örstadius et al. 2015; Wächter and Melzer 2020).

Macroscopically, the psathyrelloid basidiomata of *I.reticulopilea* enables ready discrimination from the coprinoid taxa of Psathyrellaceae. *Gasteroagaricoides* spp. have a densely granular-warty pileus and *Macrometrula* spp. have a volva (Singer 1948; Reid 1986). *Iugisporipsathyrareticulopilea* can be distinguished from these species by the smooth pileus and absence of a volva. Amongst the abundant psathyrelloid taxa of Psathyrellaceae, only the species of *Typhrasa* have slight to distinct ridge-like folds on the pileus. However, no species has a reticulate-ridged pileus similar to that of *I.reticulopilea*. In addition, the pileus surface of *I.reticulopilea* is covered by a white, inconspicuous, but persistent villus. This feature also readily distinguishes *I.reticulopilea* from known species of *Typhrasa* (Örstadius et al. 2015; Wang et al. 2021).

Microscopically, almost all species of Psathyrellaceae have smooth spores. Granulose spores are observed only in *Coprinopsis*, *Coprinellus* and *Psathyrella*, but are extremely rare. Verrucose spores are known only in *Lacrymaria*. No species has an obvious suprahilar plage as in *I.reticulopilea* (Guzmán et al. 1990; Örstadius and Knudsen 2012; Örstadius et al. 2015). In the classification system of Smith (Smith 1972), some species with ornamented spores were classified in Psathyrellasubg.Panaeolina (Maire) A.H. Smith. Those species are now excluded from the Psathyrellaceae and are classified in *Panaeolina* Maire, based on phylogenetic relationships and spores that do not fade in concentrated sulphuric acid (Kirk et al. 2013; Zhao et al. 2017). Detailed morphological comparison of *Iugisporipsathyra* and psathyrelloid genera of Psathyrellaceae is presented in Table 2.

**Table 2.** Summary of morphological characteristics used to discriminate psathyrelloid genera in the Psathyrellaceae.

	*Britzelmayria*	*Candolleomyces*	*Cystoagaricus*	*Heterospathyrella*	*Homophron*	*Iugisporipsathyra*	*Kauffmania*	*Lacrymaria*	*Olotia*	*Psathyrella*	*Typhrasa*
**Pileus surface**	smooth	smooth	fibrillose, squamulose, spiny, or squarrose; hyphae	smooth	smooth	**non-obvious villus; hyphae**	smooth	tomentose; hyphae	smooth	smooth	slight to distinct ridge-like folds
**Veil**	wipeable; hyphae	wipeable; hyphae	absent	wipeable; hyphae	absent	**absent**	wipeable; hyphae	absent	wipeable; hyphae	wipeable; hyphae, rarely subglobose cells	wipeable; hyphae
**Cap or lamellae**	non-deliquescent	non-deliquescent	non-deliquescent	non-deliquescent	non-deliquescent	**non-deliquescent**	non-deliquescent	non-deliquescent	non-deliquescent	non-deliquescent	non-deliquescent
**Spore surface**	smooth	smooth	smooth	smooth	smooth	**ridges ornamentation with obvious suprahilar plage**	smooth	often warty	smooth	smooth, rarely granulose or with myxosporium	smooth
**Basidia**	monomorphic	monomorphic	monomorphic	monomorphic	monomorphic	**monomorphic**	monomorphic	mono- to dimorphic	monomorphic	monomorphic	monomorphic
**Pseudoparaphyses**	absent	absent	absent	present	absent	**present**	absent	absent	absent	rarely present	absent
**Pileipellis**	paraderm	hymeniderm to paraderm	paraderm	hymeniderm to paraderm, covered by a 1 cell deep layer of periclinal hyphae	hymeniderm to paraderm. simple hairs sometimes present	**Hymeniderm, mixes with sparsely simple hairs**	hymeniderm to paraderm	hymeniderm	hymeniderm to paraderm	hymeniderm, paraderm, rarely cutis	hymeniderm to paraderm
**Pleurocystidia**	thin-walled	absent	thin-walled	thin-walled	thick-walled	**absent**	thin-walled	thin-walled	predominantly spatula-shaped and strongly pediculated	thin-walled or rarely slight thick-walled	thin-walled, with intracellular oily drops or globules
**Cheilocystidia**	present	present	present	present	present	**present**	present	present	present	present	present
**Pileocystidia**	present	absent	absent	absent	absent	**absent**	absent	absent	absent	very rarely present	absent

## Supplementary Material

XML Treatment for Iugisporipsathyra

XML Treatment for Iugisporipsathyra reticulopilea

## Supplementary materials

Supplementary material 1 *Iugisporipsathyrareticulopilea* gen. et sp. nov. (Agaricales, Psathyrellaceae) from Tropical China Produces Unique Ridge-ornamented Spores with an Obvious Suprahilar Plage Data type phylogenetic Explanation note A nexus file contains alignment sequence and original tree of ML and Bayes. https://binary.pensoft.net/file/704577This dataset is made available under the Open Database License (http://opendatacommons.org/licenses/odbl/1.0/). The Open Database License (ODbL) is a license agreement intended to allow users to freely share, modify, and use this Dataset while maintaining this same freedom for others, provided that the original source and author(s) are credited.Jun-Qing Yan, Yu-Guang Fan, Sheng-Nan Wang
